# Activin/Nodal signaling mediates dorsal–ventral axis formation before third quartet formation in embryos of the annelid *Chaetopterus pergamentaceus*

**DOI:** 10.1186/s13227-020-00161-y

**Published:** 2020-08-10

**Authors:** Alexis R. Lanza, Elaine C. Seaver

**Affiliations:** Whitney Laboratory for Marine Bioscience, University of Florida, Saint Augustine, USA

**Keywords:** Organizer, Dorsal–ventral axis, Spiralian, Activin/Nodal, Annelid

## Abstract

**Background:**

The clade of protostome animals known as the Spiralia (e.g., mollusks, annelids, nemerteans and polyclad flatworms) shares a highly conserved program of early development. This includes shared arrangement of cells in the early-stage embryo and fates of descendant cells into embryonic quadrants. In spiralian embryos, a single cell in the D quadrant functions as an embryonic organizer to pattern the body axes. The precise timing of the organizing signal and its cellular identity varies among spiralians. Previous experiments in the annelid *Chaetopterus pergamentaceus* Cuvier, 1830 demonstrated that the D quadrant possesses an organizing role in body axes formation; however, the molecular signal and exact cellular identity of the organizer were unknown.

**Results:**

In this study, the timing of the signal and the specific signaling pathway that mediates organizing activity in *C. pergamentaceus* was investigated through short exposures to chemical inhibitors during early cleavage stages. Chemical interference of the Activin/Nodal pathway but not the BMP or MAPK pathways results in larvae that lack a detectable dorsal–ventral axis. Furthermore, these data show that the duration of organizing activity encompasses the 16 cell stage and is completed before the 32 cell stage.

**Conclusions:**

The timing and molecular signaling pathway of the *C. pergamentaceus* organizer is comparable to that of another annelid, *Capitella teleta*, whose organizing signal is required through the 16 cell stage and localizes to micromere 2d. Since *C. pergamentaceus* is an early branching annelid, these data in conjunction with functional genomic investigations in *C. teleta* hint that the ancestral state of annelid dorsal–ventral axis patterning involved an organizing signal that occurs one to two cell divisions earlier than the organizing signal identified in mollusks, and that the signal is mediated by Activin/Nodal signaling. Our findings have significant evolutionary implications within the Spiralia, and furthermore suggest that global body patterning mechanisms may not be as conserved across bilaterians as was previously thought.

## Background

During early development, embryos become polarized either by signaling among cells or by inheritance of asymmetrically localized determinants, and an overall body plan emerges in which anterior–posterior, dorsal–ventral, and left–right axes are defined [1, 2]. In many organisms, the signaling molecules that govern body axes patterning are emitted by a cell or cluster of cells known as an embryonic organizer [3]. An embryonic organizer is a signaling center that has the capacity to induce the fates of surrounding embryonic cells and dictate the position of various tissue types [4–6].

**Fig. 1:**
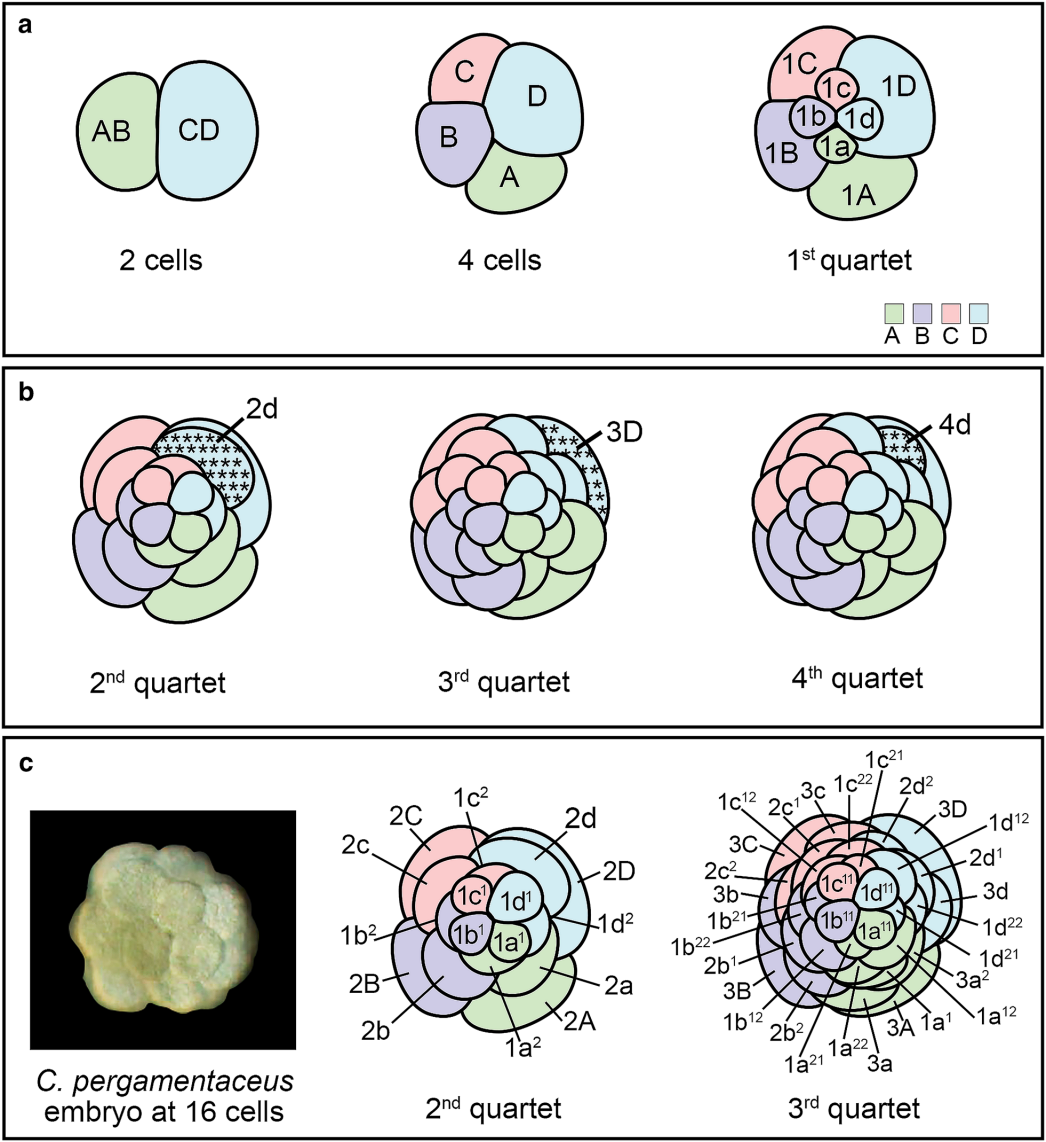
Unequal spiral cleavage. Schematics of unequally cleaving spiralian embryos during early development. All schematics are in animal view. **a** The first cleavage gives rise to two unequally sized cells (left). At second cleavage, four macromeres A (green), B (purple), C (pink) and a larger cell, D (blue) are born (center). At third cleavage, the micromeres of the 1st quartet are born (1a, 1b, 1c, 1d) (right). **b** The embryonic organizer in spiralians is typically derived from the D quadrant (cells in blue). Blue cells with asterisks mark cells that have been identified as embryonic organizers in various spiralian species (see text). Cells in green, purple, pink and blue are descendants from the A, B, C and D quadrants, respectively. **c** *C. pergamentaceus* cleavage pattern. Cropped DIC image of live *C. pergamentaceus* embryo (left). At 2nd quartet stage, there are 16 cells in the embryo, and micromeres 1d^1^ and 2d are larger than the corresponding micromeres in the other quadrants (center schematic). At 3rd quartet stage, there are 32 cells in the embryo (right schematic)

In embryos of spiralians, a group of animals consisting of mollusks, annelids, nemerteans, polyclad flatworms and several other clades, there is a highly stereotypic developmental program called spiral cleavage. This shared, conserved, cleavage program allows for the identification of individual cells, and for making intertaxonomic comparisons among homologous cells. Such comparisons are facilitated by a standard nomenclature [7]. In unequal cleaving spiralian embryos, the first cleavage division results in two unequal-sized cells called AB and a larger cell, CD (Fig. 1a, left schematic). At the second cleavage division, these cells give rise to four macromeres called A, B, C and a larger cell, D (Fig. 1a, center schematic). The macromeres define the four quadrants of the embryo and the D quadrant macromere is usually the first to divide. The subsequent daughter cells are then named relative to the four embryonic quadrants. For instance, at the third cleavage division, cells A, B, C, and D divide, and each gives rise to two daughter cells; a large macromere at the vegetal pole, and a smaller micromere toward the animal pole. The vegetal pole macromeres are named 1A, 1B, 1C, and 1D, while the micromeres are named 1a, 1b, 1c, and 1d (Fig. 1a, right schematic). These micromeres are also known as the 1st quartet micromeres. Multiple sets of quartets are ultimately produced from the macromeres, and the birth of each micromere quartet alternates between a clockwise and counterclockwise position due to shifts in the angle of the mitotic spindles in relation to the animal–vegetal pole of the embryo [8]. At the fourth cleavage division, the macromeres again give rise to two daughter cells, a larger macromere and a smaller micromere, while the 1st quartet micromeres give rise to two daughter micromeres. Therefore, macromeres 1A, 1B, 1C, and 1D divide to form macromeres 2A, 2B, 2C, and 2D and the 2nd quartet micromeres 2a, 2b, 2c, and 2d, respectively. At the same time, the 1st quartet micromeres 1a, 1b, 1c, and 1d also divide, and give rise to cells 1a^1^, 1b^1^, 1c^1^, and 1d^1^ and 1a^2^, 1b^2^, 1c^2^, and 1d^2^, respectively. Micromeres with a “1” superscript are positioned closer to the animal pole than those denoted with a “2” superscript. A similar spiral cleavage pattern is followed through to the onset of gastrulation.

In early cleavage stages of spiralian embryos, the D quadrant typically gives rise to a single cell with organizing activity, which functions to pattern the body axes. In equal cleaving forms, the specification of the D quadrant occurs by induction, and the timing of induction often closely precedes the action of the organizer, sometimes within the same cell cycle [9, 10]. In contrast, in unequal cleaving spiralians, the identity of the D quadrant is visibly apparent as the largest cell at the four cell stage, several cell cycles prior to the requirement for the organizer signal. While the precise onset of the organizing activity signal is experimentally difficult to establish, the timing of its completion has been determined in several species. Both the timing and the cellular localization of the signal vary. For example, the earliest completion of organizing activity reported to date occurs in the annelid *Capitella teleta.* In *C. teleta*, the requirement for organizing activity is completed during the 2nd quartet, when embryos typically have 16 cells, and is localized to micromere 2d (Fig. 1b, left schematic) [11]. In mollusks such as the mud snail *Trita obsoleta* (formerly *Ilyanassa obsoleta*), and the limpet *Patella vulgata*, organizing activity is completed when the 3rd quartet of micromeres is present, when the embryos typically have 24 cells, and is localized to macromere 3D (Fig. 1b, center schematic) [12–17]. In the slipper shell snail *Crepidula fornicata,* organizing activity is completed even later, when the 4th quartet of micromeres is present. Organizing activity is localized to micromere 4d, a cell that is typically born at the 25 cell stage (Fig. 1b, right schematic) [18–20]. It is further reported that in the oligochaete *Tubifex tubifex*, organizing activity is completed during the 4th quartet, when embryos typically have 22 cells, and is localized to not one, but two cells named 2d^11^ and 4d [21].

**Fig. 2:**
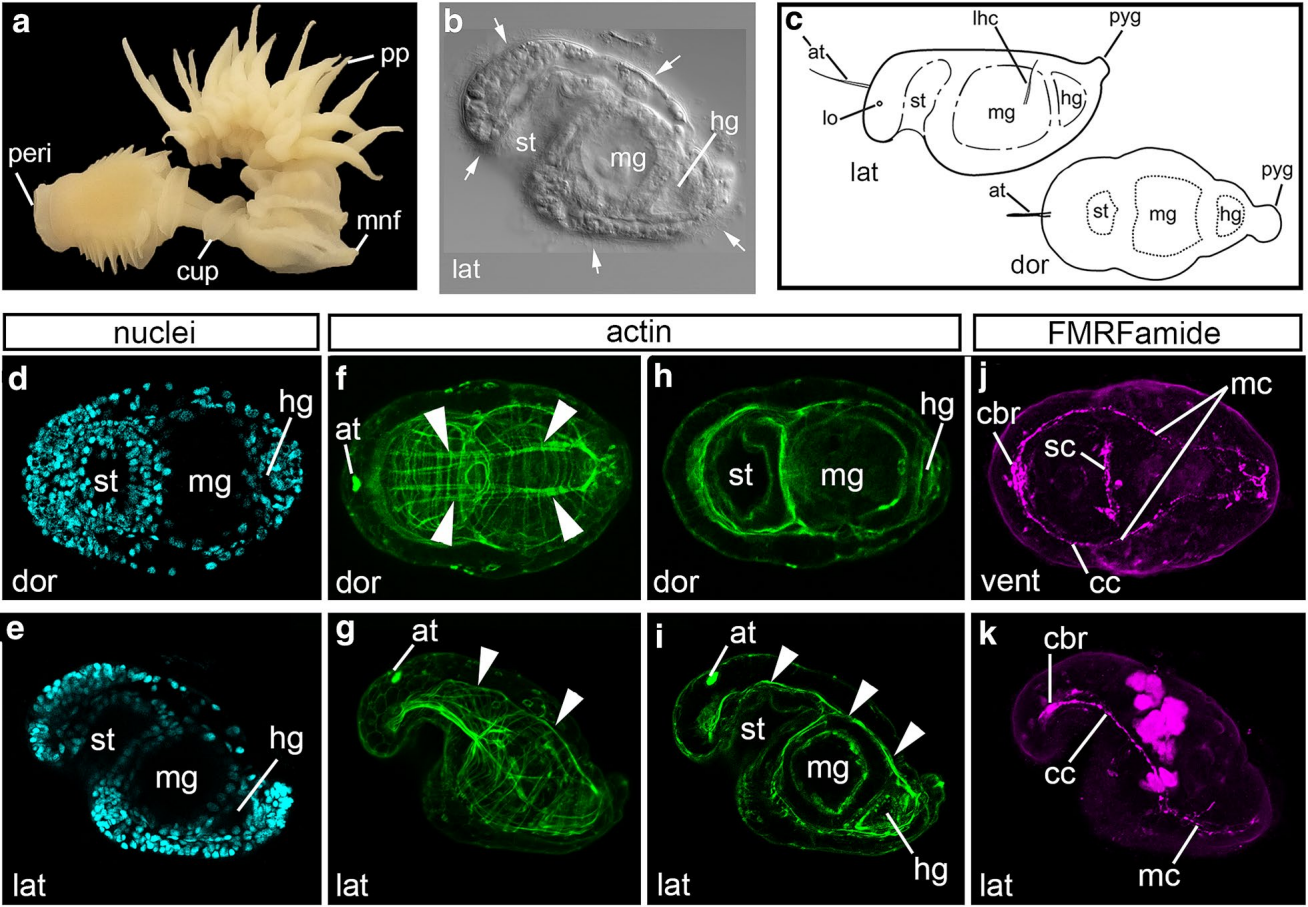
Morphology of *C. pergamentaceus.* Anterior is to the left in all panels. **a** Adult. **b** DIC image in fixed L2 larva (lateral view). **c** Schematics depict lateral (left) and dorsal (right) views of larvae at L2 stage, approximately 36 h post-fertilization. Schematics adapted from [29]. **d**–**j** are of a single L2 larva in dorsal view and **e**–**k** are of a second L2 larva in lateral view. **d**–**k** are merged confocal-stacked images of larvae labeled for nuclei with Hoechst (**d**, **e**), actin filaments with phalloidin (**f**, **i**), and neurons with anti-FMRFamide (**j**, **k**). Stacks of confocal micrographs of actin staining are displayed at two different depths in the body to show muscles (**f**, **g**, **i**) and the tripartite gut (**h**, **i**). at: apical tuft attachment point; cc: circumesophageal connective nerve; cbr: cerebral commissure; cup: cupule; dor: dorsal; mnf: modified neuropodia fans; hg: hind gut; lat: lateral; lhc: lateral hooked cilia; lo: larval ocellus; mc: main connective; mg: midgut; peri: peristomium; pp: posterior parapodia; pyg: pygidium; sc: subesophageal commissure; st: stomodeum; white arrows: cilia; white arrowheads: dorsal longitudinal muscle fibers

The identity of the organizer is currently unknown in the early branching annelid *Chaetopterus pergamentaceus* Cuvier, 1830 [22, 23]. Also called parchment tube worms, *C. pergamentaceus* are filter feeders that live in U-shaped tubes buried beneath the surface of coastal habitats (Fig. 2a) [24]. They are amenable to *in vitro* fertilization, and their embryological development has previously been described [25–28]. Following fertilization, the first cleavage is unequal and each subsequent cleavage division occurs at intervals of 15–20 min at 22 °C (Fig. 1c, left image). Cells undergo almost synchronous divisions through to the 32 cell stage [28]. At the 16 cell stage, micromeres 1d^1^ and 2d are larger than their sister micromeres (Fig. 1c, center schematic) [27]. All 16 cells then divide, almost synchronously, to give rise to a 32 cell stage embryo in which the 3rd quartet cells are of similar size (Fig. 1c, right schematic). The transition from the 32 to 64 cell stage is less uniform. While the D macromere divides soon after the completion of the 32 cell stage, other cells in the embryo are slower to divide [28]. Embryos become ciliated around the gastrula stage, and later give rise to larval forms [27].

A *C. pergamentaceus* larval staging system was previously described by Irvine et al. [29]. Embryos give rise to L1 larvae at 18 h post-fertilization at 22 °C, and by 36 h post-fertilization, larvae are at the L2 stage. At L2, larvae possess several morphological features that enable the clear detection of an anterior–posterior axis, a dorsal–ventral axis, and bilateral symmetry (Fig. 2b) [28, 29]. These larvae are approximately 180 × 90 μm in size, possess two larval ocelli (Fig. 2c; lo), and develop ciliary structures such as an anterior apical tuft (Fig. 2c; at) and a pair of lateral hooked bristles (Fig. 2c; lhc) [29]. In addition to these specialized and visually distinct ciliated structures, the larval ectoderm surface is covered with cilia (Fig. 2b). L2 larvae actively feed, and their gut has a tripartite organization with visually distinct gut compartments. At its anterior end is the stomodeum, which opens to the exterior on the ventral surface of the body (Fig. 2b, c; st). The stomodeum is connected to the large and medially positioned midgut (Fig. 2b, c; mg) through a narrow junction, and posterior to the midgut is the hindgut, which exits the body just anterior to the pygidium (Fig. 2b, c; hg). Development of the larval gut has previously been characterized [30].

Previous experimental investigations in *C. pergamentaceus* have demonstrated that the D quadrant possesses organizing activity [27, 31]. Blastomeres isolated from embryos at the 2 cell stage indicated that cells AB and CD have different developmental potential [27]. Although both isolates develop into partial larvae, only larvae resulting from CD isolates form an eye. Lineage tracing of blastomeres at the 4 cell stage determined that the left and right larval eyes are derived from the A and C macromeres, respectively [31]. In most other spiralians, the left and right larval eyes are derived from cells 1a and 1c, descendants of the A and C macromeres [10, 32, 33]. Therefore, although cell AB has the potential to form an eye, its formation seems to be dependent upon an inductive signal from cells derived from the D quadrant [27].

The organizing role of the D quadrant in *C. pergamentaceus* was further demonstrated through twinning experiments [31]. Uncleaved zygotes were lightly compressed with a glass cover slip to equalize the first cleavage division. Instead of the normal pattern of division into two unequal-sized cells, AB and a larger CD cell, the pressure of the cover slip forced the equal distribution of cytoplasmic determinants across both cells. This resulted in two equally sized cells, both with CD identity. These divided into four cell stage embryos that possessed two larger, presumptive D cells, and two C cells [31]. Four cell stage embryos in which both D quadrants were located diagonally to each other developed into larvae with two trunks, a shared mouth, and several other duplicated structures. Furthermore, bilateral symmetry and dorsoventral polarity were distinguishable in these twinned larvae [31]. These results suggest that the D quadrant not only possesses the ability to influence the C quadrant into contributing to the formation of a second body axis, but is also able to establish dorsal–ventral polarity of the newly induced tissues. Together, both studies sufficiently demonstrate the presence of organizing ability in *C. pergamentaceus*, specifically the ability of the D quadrant to influence the fate of other cells, and induce a second body axis.

Investigations into the identity of the molecular signals that mediate organizing activity in spiralians have revealed variation among species. In mollusks, activation of the MAPK pathway has been linked to organizing activity in *T. obsoleta* [17], *Tectura scutum* [14], and *Haliotis asinina* [34], as well as to the specification of the organizer in *C. fornicata* [18]. However, in the annelids *Platynereis dumerilii* [35], and *C. teleta* [11], MAPK has no detectable function in axis specification. Besides MAPK signaling, studies in *T. obsoleta* [36] and *Crassostrea gigas* [37] have provided experimental support to suggest that the BMP signaling pathway functions in dorsal–ventral axis patterning. Interestingly, in *C. teleta,* BMP signaling does not have a primary function in organizing activity; rather it is the Activin/Nodal pathway, a sister branch to BMP signaling, that is required to pattern the dorsal–ventral axis [38, 39].

In this study, we investigate the molecular mechanisms driving axis specification in the early branching annelid *C. pergamentaceus*. Through short exposures of early-stage embryos to chemical inhibitors, we investigate both the molecular identity and timing of organizing activity during early development. Since *C. pergamentaceus* is an early branching annelid, our findings provide phylogenetically relevant insights of the ancestral mechanism for dorsal–ventral axis patterning in Annelida.

## Methods

### Animal care and in vitro fertilization

Live adult *C. pergamentaceus* were obtained from the Marine Resources Center at Marine Biological Laboratory (MBL) in Woods Hole, MA and maintained in tanks of running sea water. The Woods Hole species of *Chaetopterus* has been identified as *C. pergamentaceus* Cuvier, 1830 using genetic data [40]. Embryos were obtained through in vitro fertilization procedures as previously outlined by Henry (1986). Adults were partially removed from their tubes using a pair of forceps and surgical scissors. Sexually mature male and female *C. pergamentaceus* are easily identifiable via the color of gametes visible through the wall of posterior parapodia [29]. In females, these segments appear yellow if filled with mature oocytes, and in males they appear white if filled with sperm. For fertilizations, a single parapodium was cut from the female and its contents were sifted through a 150-µm Nitex sieve with filtered seawater (FSW) into a 60-mm petri dish. This removed mucus and tissue debris. Oocytes were rinsed 3× with FSW to further remove mucus and then left for 20 min in FSW to trigger egg activation via the breakdown of germinal vesicles. Typically, thousands of eggs can be collected from a single parapodium. Sperm was collected during the 20 min egg activation interval. A single parapodium was cut from a male, and approximately 150 µl of sperm was collected using a 1-ml transfer pipette into a 1.5-ml Eppendorf tube. Sperm was diluted at a 1:1000 sperm:FSW ratio. Sperm became motile after about 5 min in FSW. Motility could be visually verified by observing a drop of diluted sperm on a glass slide using a compound microscope. To initiate fertilization, eggs were swirled into the middle of the petri dish and a single drop of motile sperm was added to the center, and then the dish left undisturbed. The eggs were exposed to sperm for 10 min, followed by 3× FSW washes to remove excess sperm and prevent polyspermy. Zygotes were monitored for first cleavage, which typically occurred 1 h post-fertilization at 22 °C. Subsequent cleavages occurred every 15–20 min at 22 °C.

### Chemical inhibition

For chemical exposure experiments, embryos from a single fertilization were aliquoted for use at each time interval, and divided into experimental and control conditions. Control embryos were exposed to Dimethyl sulfoxide (DMSO) as a solvent control, while experimental embryos were exposed to a pathway-specific chemical inhibitor. Embryos from a single fertilization were used in one time series experiment, and this is defined as a single technical replicate. Approximately 100 embryos were used per replicate.

Activin/Nodal signaling was inhibited using SB431542 (Santa Cruz; Cat No: SC-204265A), a chemical inhibitor that prevents the phosphorylation of the Activin/Nodal type 1 receptors ALK4, ALK5, and ALK7. BMP signaling was inhibited using two different chemical inhibitors, DMH1 (Sigma; Cat No: D8946), an inhibitor of ALK2, a BMP type 1 receptor, and dorsomorphin dihydrochloride (Tocris; Cat No: 3093), an inhibitor of both ALK2 and ALK3 BMP type 1 receptors. The ERK/MAPK pathway was inhibited using U0126 (Sigma, Saint Louis, MO), an inhibitor of MEK1 and MEK2. Each drug was resuspended in 100% DMSO to a 10-mM stock, and was further diluted in filtered seawater (FSW) to working concentrations. To monitor for potential toxic effects of the DMSO solvent, control embryos were exposed to DMSO in FSW at a concentration equivalent to the experimental condition (0.05–0.5% DMSO).

Initial experiments included exposure of early-stage embryos to a range of inhibitor concentrations between 5 and 50 µM. Exposures above 20 μM with all drugs tested resulted in arrested development. Detailed analysis of exposure to SB431542 was performed at a concentration of 20 μM in FSW. Exposure to SB431542 was conducted during the following time intervals: 4–32 cell stage; 8–32 cell stage; 16–32 cell stage; 32 cell to late cleavage stage; late cleavage to early gastrula stage. Embryos were visually monitored with a dissecting microscope for the birth of each quartet. Only when embryos were at the appropriate stage they were individually pipetted into or out of a petri dish prefilled with 3 ml of the appropriate chemical inhibitor. This allowed us to make fine adjustments to any variation in the timing of cell division among embryos. In *C. pergamentaceus,* the D quadrant is the first to divide, and so micromeres of the D quadrant are born first [27]. Furthermore, in *C. pergamentaceus*, all cells divide synchronously up to the 32 cell stage [28].

Embryos exposed to 20 μM SB431542 during the interval between the 8 and 32 cell stage did not survive development past larval stage L1 to larval stage L2 (data not shown; 4 technical replicates, approximately 100 embryos used per replicate). As such, deductions could not be made about the effects of Activin/Nodal inhibition during this time interval. Dorsomorphin dihydrochloride, DMH1, and U0126 were all used at a concentration of 20 μM in FSW. Exposures with these drugs were conducted for 90 min time intervals at the following cell stages: 4 cell to late cleavage stage; late cleavage to early gastrula stage.

Following exposure to inhibitors, embryos were washed with FSW for 4× 1 min, followed by 4× 15 min to remove drug or DMSO. Embryos were raised for approximately 72 h to larval stage 2 (L2) at room temperature (22 °C) in FSW with 60 μg/ml penicillin (Sigma-Aldrich Co., St Louis, MO, USA) and 50 μg/ml streptomycin (Sigma-Aldrich Co., St Louis, MO, USA). Specimens were fixed for immunolabeling as described below.

### Immunolabeling

Larvae were exposed to a 1:1 dilution of 0.37 M MgCl_2_:FSW for 10 min to prevent larval muscle contractions upon fixation. Larvae were then fixed using 3.7% paraformaldehyde (PFA) in FSW for 30 min. Fixation was terminated via 2× quick exchanges with phosphate-buffered saline (PBS), followed by 2× washes in PBS with 0.1% Triton X-100 (PBT). Animals were stored in PBS at 4 °C for up to 1 month, whereby immunolabeling and phalloidin staining were conducted.

Fixed animals were placed in a 3-well depression glass spot plate, and treated with a blocking solution of PBT + 10% heat-inactivated goat serum (Cat No. G9023, Sigma-Aldrich Co., US) for 1 h at room temperature (RT). Primary antibody diluted in blocking solution was added to samples and incubated at 4 °C overnight (O/N). The primary antibody was a rabbit anti-FMRFamide antibody used at 1:800 dilution (Cat No: 20091, Immunostar). Following incubation, the primary antibody was removed via five washes with PBT over the course of an hour. Specimens were incubated a 1:400 dilution of goat anti-mouse secondary antibody conjugated with the fluorescent tag Alexa Fluor 488 (Cat No. A11001, Molecular Probes) for 2 h at RT. Following incubation, unbound secondary antibody was removed via five washes with PBT over the course of an hour. Larval muscles were visualized by staining with a 1:200 dilution of Alexa Fluor 488-phalloidin (Cat No. A12379, Thermo Fisher Scientific, US) for 2 h at RT, then washed five times with PBT over the course of an hour. Specimens were equilibrated in 80% glycerol in 1 × PBS plus 0.125 μg/ml Hoechst, a DNA visualization stain.

### Microscopy, scoring, and imaging

The fixed experimental and control animals in each well of the plate were observed at low magnification for consistency of the phenotypes within a sample. For detailed phenotypic analysis, a random subset of the resulting specimens was placed on microscope slides and viewed from multiple orientations to analyze features that indicate the presence of an anterior–posterior axis, dorsal–ventral axis and bilateral symmetry. Only specimens examined in this level of detail were scored and formally counted in the sample size. Following fluorescent staining or immunohistochemistry, animals were imaged using an LSM 710 confocal microscope (Zeiss). To determine the position of various structures within the body, scans through the entire larval body were taken. 3D reconstructions were generated using Fiji (Fiji Is Just ImageJ) [41], and images were processed in Adobe Photoshop CC (version 2017.1.1). Image of the live 16 cell embryo in Fig. 1c (left) was closely cropped and put on a solid background.

### Statistical analysis

Each larva analyzed was categorized according to treatment groups: DMSO control, U0126 during 4 cell to late cleavage stage, U0126 during late cleavage to early gastrula stage, DMH1 during 4 cell to late cleavage stage, DMH1 during late cleavage cleave to early gastrula stage, dorsomorphin dihydrochloride during 4 cell to late cleavage stage, dorsomorphin dihydrochloride during late cleavage stage to early gastrula stage, SB431542 during 4–32 cell stage, SB431542 during 16–32 cell stage, SB431542 during 32 cell to late cleavage stage, SB431542 during late cleavage to early gastrula stage. For statistical analyses, larval phenotypes were sorted into one of two categories: dorsal ventral axis detected or no dorsal ventral axis detected. To determine which of the conditions differ from each other, an omnibus Chi square test of homogeneity was performed, followed by post-hoc pairwise comparisons using a *z* test of two proportions. Bonferroni correction was applied to account for multiple comparisons.

## Results

### *Chaetopterus pergamentaceus* body plan

We analyzed organ system organization and differentiated tissue types in *C. pergamentaceus* larvae by labeling for nuclei with Hoechst (Fig. 2d, e), muscle fibers (Fig. 2f, g) and cell cortices (Fig. 2h, i) with phalloidin, and neuronal processes with the cross-reactive antibody, anti-FMRFamide (Fig. 2j, k). Specifically, the distinct stomodeum, midgut, and hindgut compartments are visible in animals labeled for nuclei (Fig. 2d, e) and filamentous actin (Fig. 2h, i). Phalloidin staining also allows for the visualization of the larval body wall musculature. L2 larvae have numerous circumferential and longitudinal muscle fibers throughout the body. Notably, there are two prominent, bilaterally symmetric longitudinal muscles that straddle the dorsal midline and span the length of the body (Fig. 2f, g, i; white arrow heads). Also visible by actin staining is the position of the apical tuft, and its attachment point is clearly located on the dorsal, anterior surface of the head (Fig. 2f, g, i). Actin labeling is similarly used to identify the attachment point of the apical tuft in the larvae of nemerteans [42–44]. In the head of *C. pergamentaceus* larvae, there is a bilateral pair of cerebral ganglia visible via the spatial expression of *COE*; a transcription factor involved in neural specification [45]. We further characterized the organization of the nervous system by visualizing a subset of nerves using an anti-FMRFamide antibody. There is a medial cluster of FMRFamide immunoreactive cells positioned between the cerebral ganglia (Fig. 2j, k; cbr). From the cerebral commissure, a pair of neuronal processes circumvents the stomodeum as the circumesophageal connective (Fig. 2j, k; cc). The pair of anti-FMRFamide immunoreactive processes is visible along the length of the ventral side of the trunk as the main connective (Fig. 2j, k; mc) and terminates in the pygidium. The two longitudinal connectives are positioned closer together in the midbody and posterior end relative to their lateral position in the head and anterior portion of the trunk. This more medial position of the connectives begins at the approximate position of the posterior face of the midgut. A subesophageal commissure is also visible, just posterior to the stomodeum (Fig. 2j; sc). These features are bilaterally symmetric and mark the ventral side of the larva. In addition, the subesophageal commissure and the FMRFamide immunoreactive cells in the head mark the anterior of the larva.

Together, the anterior apical tuft, stomodeum and cerebral commissure, along with the posterior hindgut, are all morphological features that enable the detection of an anterior–posterior axis. The dorsal position of both the apical tuft attachment point and the pair of longitudinal muscles, along with both the ventral stomodeum opening and ventral neuronal processes, likewise indicate a clear dorsal–ventral axis. Although L2 larvae have bilaterally symmetric eye spots, we could not reliably detect them since exposure to detergents during the antibody labeling process causes the loss of eye pigment. Bilateral symmetry is therefore confirmed by the presence of the pair of ventral neuronal processes and the pair of dorsal longitudinal muscles.

### Inhibition of the ERK/MAP kinase pathway results in abnormal gut and muscle formation

**Fig. 3:**
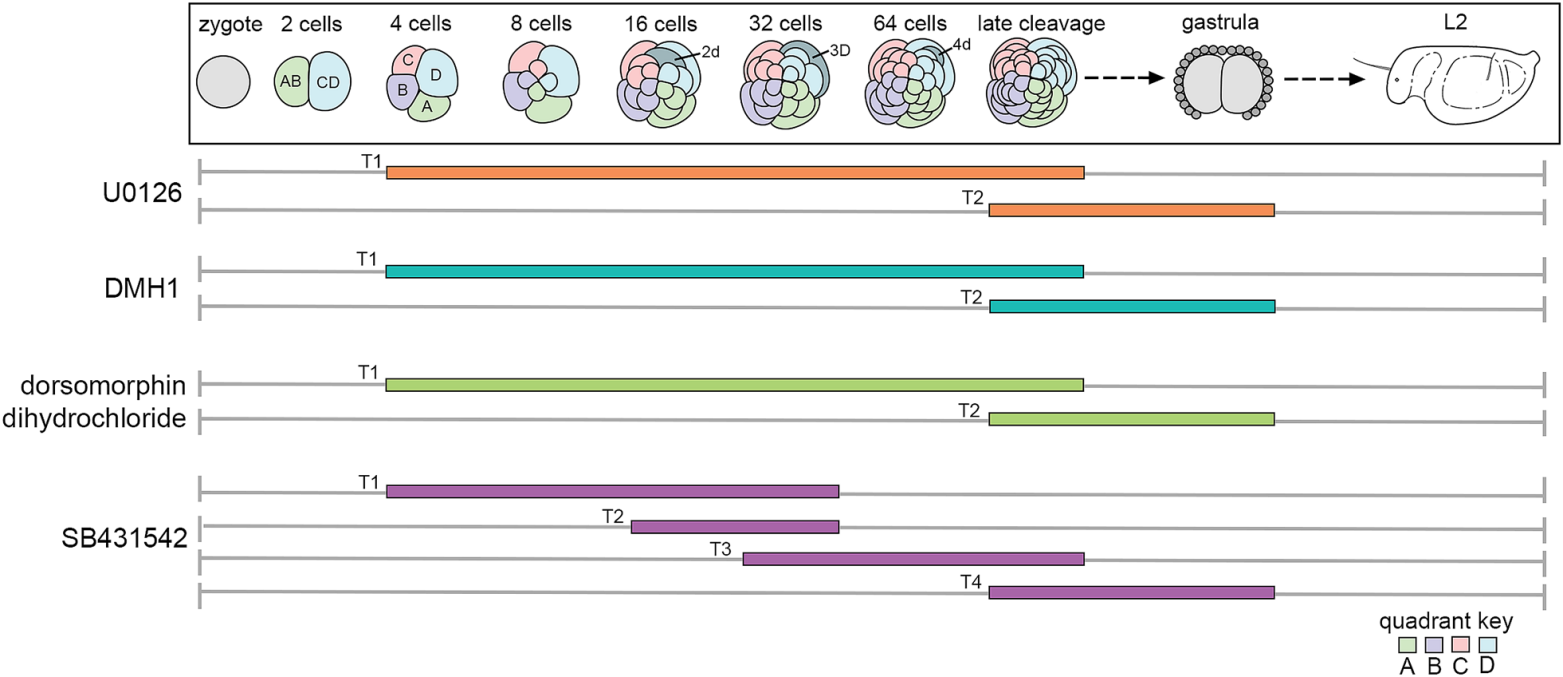
Experimental design for drug exposure experiments. Diagram depicts the early developmental stages of *C. pergamentaceus* from zygote to larval stage L2 (larval schematic adapted from [29]). Drug exposures were conducted during multiple time intervals. Exposures to U0126 (orange bars), DMH1 (teal bars), and dorsomorphin dihydrochloride (green bars) were conducted during the 4 cell to late cleavage stage (T1), and the late cleavage stage to early gastrula time intervals (T2). Exposures to SB431542 (purple bars) were conducted during the 4–32 cell (T1); 16–32 cell (T2); 32 cell to late cleavage stage (T3); and late cleavage stage to early gastrula (T4) time intervals. After removal of the chemical inhibitor, embryos were raised in sea water for approximately 72 h to L2 larvae, and then fixed for phenotypic analysis (vertical gray line on right hand side). Horizontal gray lines indicate duration of the experiment. Cells labeled as 2d, 3D, and 4d have been identified as organizers in other spiralians (see text)

To investigate the identity and timing of the signaling pathway involved in axis specification in *C. pergamentaceus*, we exposed embryos at early cleavage stages to small chemical inhibitors of specific signaling pathways. Experiments were performed at various time intervals and drug concentrations (Fig. 3). Initial treatments included exposure to each drug at concentrations ranging from 5 to 50 μM. The lowest concentration at which there was a consistently reproducible larval phenotype was selected for detailed analysis. Exposures above 20 μM with any drug tested resulted in arrested development. For all experiments, embryos were exposed to concentrations of DMSO equivalent to experimental conditions to control for nonspecific effects of the solvent.

Experimental evidence showing that activation of the ERK/MAPK pathway is associated with patterning of the dorsal–ventral axis of some spiralians led us to investigate the role of this pathway during early development in *C. pergamentaceus* using the small molecule inhibitor U0126. U0126 functions by inhibiting the kinase activity of both MEK1 and MEK2 [46]. As a result, MEK1/2 is unable to phosphorylate and activate MAPK, and thereby inhibits the ERK/MAP kinase pathway. In experiments investigating the effects of inhibiting the ERK/MAP kinase pathway with U0126, exposures were conducted during two independent time intervals: the 4 cell to late cleavage stage and from late cleavage stage to early gastrula (Fig. 3). The duration of each time interval was 90 min, during which approximately five cleavage divisions occurred. Following drug exposure, embryos were raised in sea water to larval stage L2, and then scored for axial anomalies.

**Fig. 4:**
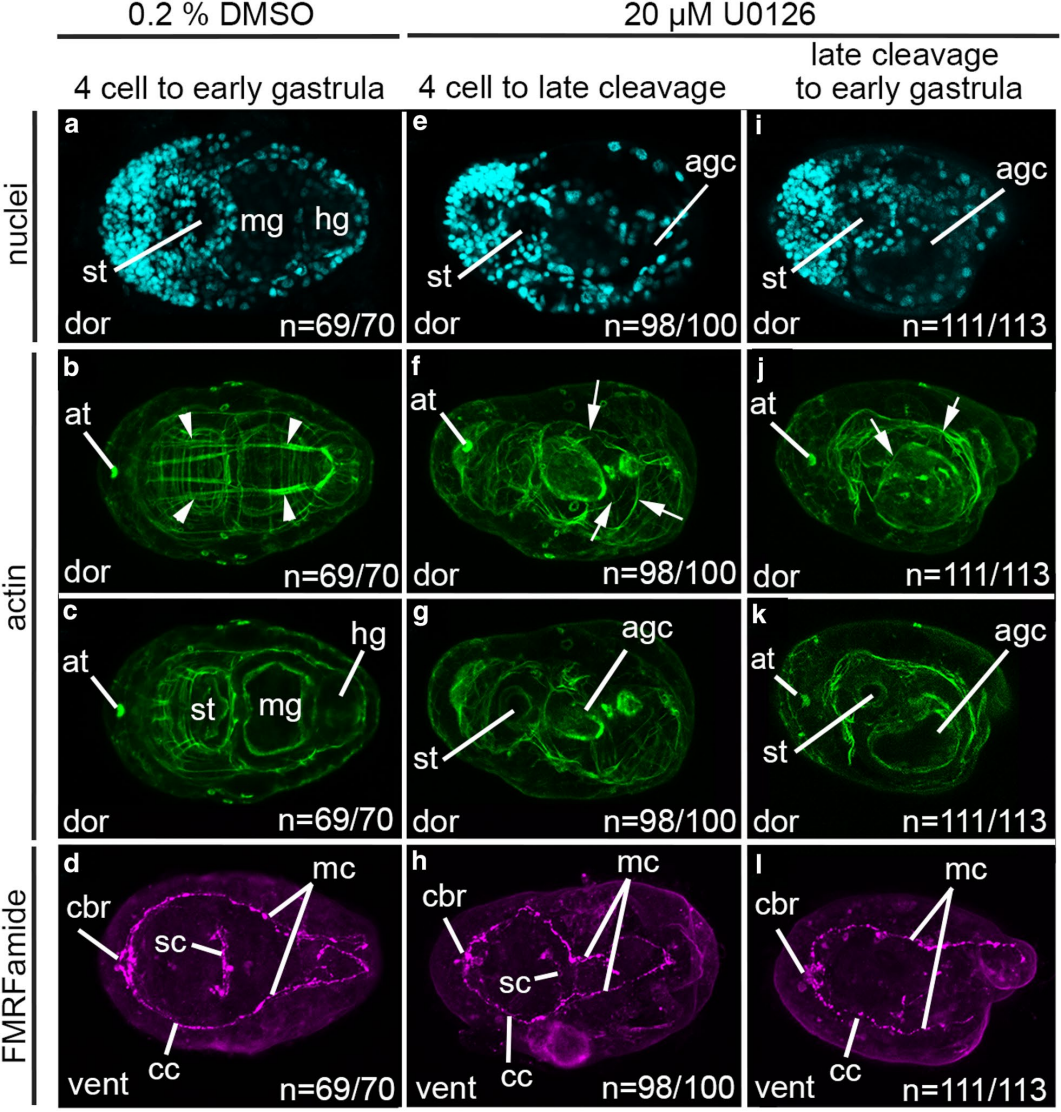
Larval phenotypes resulting from early embryonic exposure to U0126. Each column contains merged confocal-stacked images from a single L2 larva. Each row depicts labeling for nuclei with Hoechst, actin filaments with phalloidin, or neurons with anti-FMRFamide. Stacks of confocal micrographs of actin labeling are displayed at two different depths in the body to show both muscles and gut cavity organization. **a**–**d** are of a control larva exposed to 0.2% DMSO during the interval between the 4 cell stage and the early gastrula. **e**–**l** are larvae resulting from exposure to 20 μM U0126. **e**–**h** depict a larva resulting from exposure during the interval between the 4 cell to late cleavage stage. **i**–**l** depict a larva resulting from exposure during the interval between late cleavage stage and early gastrula. All panels are dorsal views with anterior to the left. agc: abnormal gut cavity; at: apical tuft attachment point; cc: circumesophageal connective; cbr: cerebral commissure; dor: dorsal; hg: hindgut; mc: main connective; mg: midgut; sc: subesophageal commissure; st: stomodeum; white arrowhead: dorsal longitudinal muscle fibers; white arrows: disorganized muscle fibers

Embryos exposed to 0.2% DMSO during the interval between the 4 cell stage and the early gastrula stage result in phenotypically normal larvae (Fig. 4a–d; *n* = 3 technical replicates). These larvae have differentiated cell types, and were morphologically analyzed following visualization of nuclei, filamentous actin, and anti-FMRFamide immunoreactive cells. Identifiable anterior features such as the stomodeum (Fig. 4a and c), apical tuft attachment point (Fig. 4b and c), and the cerebral commissure (Fig. 4d) are present. A posterior hindgut is also present (Fig. 4a and c). A dorsal–ventral axis is detectable via the presence of the dorsal position of the apical tuft attachment point (Fig. 4b and c) and the pair of dorsal longitudinal muscles (Fig. 4b), while ventral is identifiable by the presence of a stomodeum, and ventral neuronal processes (Fig. 4d). Bilateral symmetry is also detectable by the presence of the bilateral pair of dorsal longitudinal muscles (Fig. 4b), and the pair of ventral neuronal processes circumventing the stomodeum and midgut (Fig. 4d). All three body axes were detectable in 99% (*n* = 69/70) of larvae.

Larvae resulting from exposure to 20 μM U0126 during the 4 cell to late cleavage stage (Fig. 4e–h) are phenotypically abnormal, but have differentiated cell types and possess all three body axes (*n* = 3 technical replicates). Anteriorly, the stomodeum (Fig. 4e and g), apical tuft attachment point (Fig. 4f), and cerebral commissure (Fig. 4h) are present. However, a gut with a clear tripartite organization is not visible, and a hindgut is not detectable (Fig. 4e and g). Instead, larvae exhibit an abnormal gut cavity in place of a midgut (Fig. 4e–g; agc). The dorsal apical tuft attachment point (Fig. 4f) along with the ventral opening of the stomodeum (Fig. 4e, g), and the ventral pair of circumesophageal and main connectives (Fig. 4h) indicate presence of a dorsal–ventral axis. Dorsal longitudinal muscles are not detected and there is a general disorganization of the muscle fibers (Fig. 4f and g). Bilateral symmetry is seen via the ventral pair of circumesophageal and main connectives circumventing the stomodeum and abnormal gut cavity (Fig. 4h). Altogether, despite abnormalities in gut formation and muscle organization, an anterior–posterior axis, dorsal–ventral axis, and bilateral symmetry are detectable in 98% (*n* = 98/100) of the resulting larvae.

Similarly, larvae resulting from exposure to 20 μM U0126 during the interval between late cleavage stage and early gastrula exhibit abnormal phenotypes and have differentiated cell types and possess all three body axes (Fig. 4i–l) (*n* = 3 technical replicates). The anterior stomodeum (Fig. 4i, k), apical tuft attachment point (Fig. 4j and k), and cerebral ganglia (Fig. 4l) are all present. These larvae exhibit a single abnormal gut cavity in place of a midgut (Fig. 4i and k), and there is no detectable hindgut (Fig. 4i and k). The dorsal apical tuft attachment point (Fig. 4j and k), together with the ventrally opened stomodeum (Fig. 4i, k) and the ventral pair of circumesophageal and main connectives (Fig. 4l) indicate the presence of a dorsal–ventral axis. Notably, actin fibers are generally disorganized, and there are few muscles present (Fig. 4j). The ventral pair of circumesophageal and main connectives circumventing the stomodeum and abnormal gut cavity (Fig. 4l) indicates bilateral symmetry. Altogether, all three body axes are detectable in 98% (*n* = 111/113) of resulting larvae.

An omnibus Chi square test of homogeneity was used to compare the proportions of larvae with and without a dorsal–ventral axis resulting from experimental and control conditions. Our analysis shows no statistically significant difference (*p* > 0.05) in the proportions of larvae with and without a dorsal–ventral axis following U0126 exposures at either developmental interval in comparison to those larvae resulting from DMSO control conditions. These results indicate that exposure to U0126 prior to gastrulation does not affect dorsal–ventral axis formation.

### BMP inhibition with DMH1 or dorsomorphin does not affect axes formation

BMP signaling has been shown to mediate organizing signaling and dorsal–ventral axis formation in some mollusks, a sister taxa to annelids [36, 37]. Since *C. pergamentaceus* is an early branching annelid, we were interested in investigating whether *C. pergamentaceus* uses the same molecular signal to mediate organizing activity as mollusks. We inhibited the BMP signaling pathway using two different chemical inhibitors, DMH1 and dorsomorphin dihydrochloride (Fig. 3). The chemical inhibitor DMH1 functions by preventing the phosphorylation of the BMP type 1 receptor ALK2 [47]. Similarly, dorsomorphin dihydrochloride functions by preventing the phosphorylation of BMP type I receptors, ALK2 and ALK3 [48].

Larvae resulting from exposure to 20 μM DMH1 (*n* = 3 technical replicates) during the interval between either the 4 cell to late cleavage stage (*n* = 78/81) or the late cleavage to early gastrula stage (n = 89/95) are phenotypically normal with no detectable abnormalities (Additional file 1: Figure S1E–L). Likewise, larvae resulting from exposure to 20 μM dorsomorphin dihydrochloride (n = 3 technical replicates) during either the 4 cell to late cleavage stage (*n* = 73/74) or the late cleavage to early gastrula stage (n = 115/117) are also phenotypically normal (Additional file 1: Figure S1M–T). There is no statistically significant difference (*p* > 0.05) between larvae exposed to DMH1 or dorsomorphin dihydrochloride and control larvae exposed to DMSO (omnibus Chi square test of homogeneity). As these exposures do not elicit detectable phenotypic effects in larvae, these inhibitors cannot be confirmed to be effective at inhibiting BMP signaling in *C. pergamentaceus.*

### Inhibition of Activin/Nodal signaling results in abnormal axial development

The Activin/Nodal branch of the TGF-beta signaling pathway mediates dorsal–ventral axis patterning in the annelid *C. teleta*, [38, 39]. To determine if Activin/Nodal signaling also mediates dorsal–ventral axis patterning in *C. pergamentaceus*, we used the small molecule inhibitor SB431542, which functions by inhibiting the phosphorylation and activation of Activin/Nodal type I receptor, ALK4/5/7 [49]. Initial experiments indicated that exposures to 20 μM SB431542 during the interval between the 4 cell to late cleavage stage but not the late cleavage to early gastrula stage resulted in larvae with axial defects (data not shown). Therefore, subsequent drug exposure experiments were conducted to more accurately determine the timing of axis specification (Fig. 3). The original time interval (4 cell to late cleavage stage) was shortened by serially delaying when the inhibitor was added, therefore moving the beginning of the exposure timeframe forward one cleavage stage at a time. Specifically, the 4 cell to late cleavage time interval was subdivided as follows: 4–32 cell, 16–32 cell, and 32 cell to late cleavage stage (Fig. 3). The duration of these time intervals was approximately 45, 15, and 45 min, respectively (cleavage divisions occur at 15-min intervals). Embryos were visually monitored for the birth of each quartet prior to adding them to the inhibitor. This experimental design allowed us to be confident about the cell stage at which the inhibitor was added to the embryos, and accounts for any uncertainty regarding the time required for the chemical inhibitor to diffuse out of the embryonic tissue.

Exposures were also conducted during the late cleavage to early gastrula stage (approximately 90-min long) as a control time interval, during which anomalies in axis patterning were not expected. Embryos were exposed to concentrations of DMSO equivalent to experimental conditions to control for nonspecific effects of the solvent during the interval between the 4 cell stage and the early gastrula stage (3 h). Following drug exposure, embryos were raised to L2 larvae and morphologically analyzed.

**Fig. 5:**
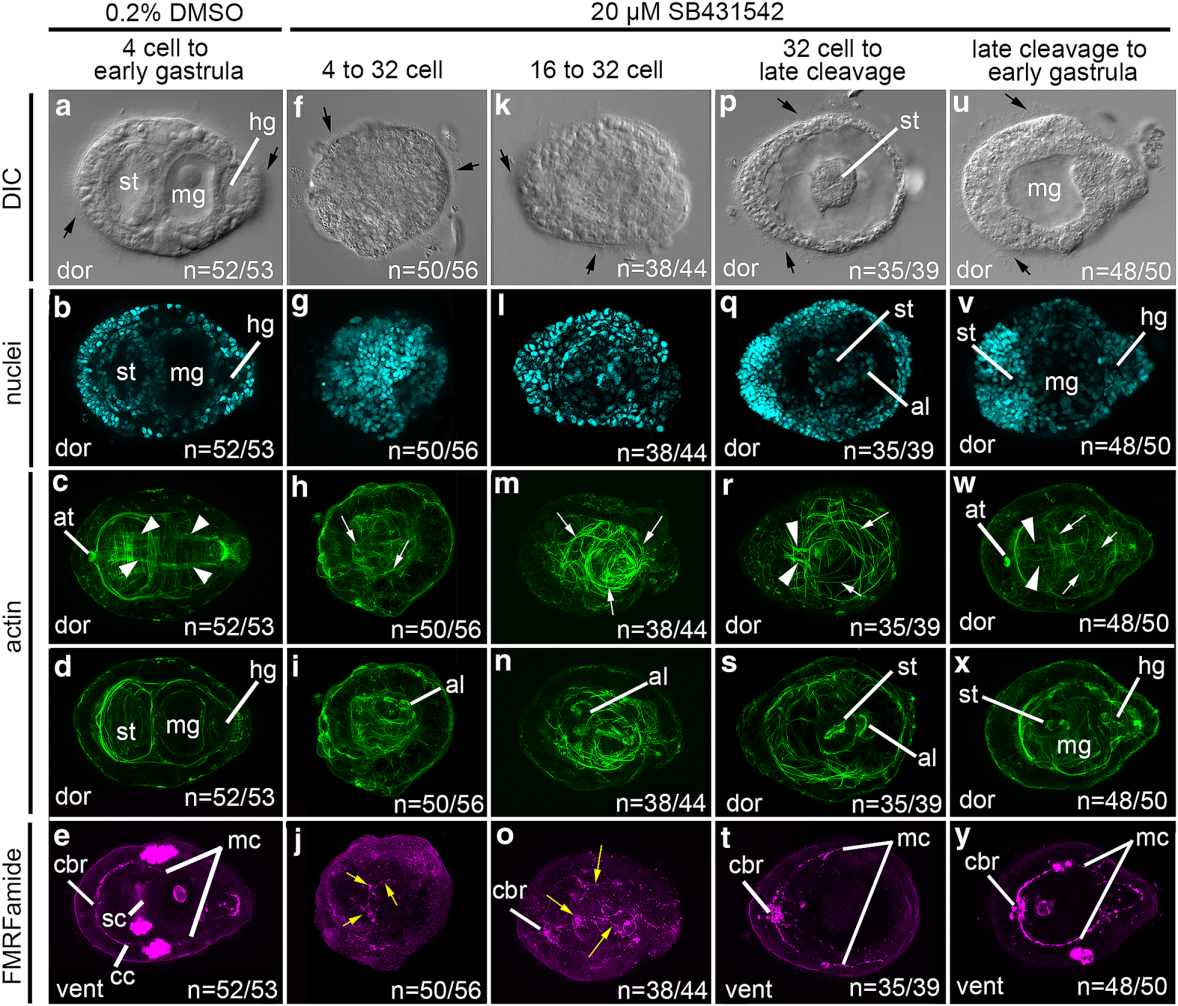
Early embryonic exposure to SB431542 affects axes formation in larvae. Each column contains images from a single L2 larva. The first row shows DIC micrographs. Fluorescent images are merged confocal stacks. **b**, **g**, **l**, **q**, **v** depict labeling for nuclei with Hoechst, **c**, **d**, **h**, **i**, **m**, **n**, **r**, **s**, **w**, **x** show actin filaments with phalloidin, and **e**, **j**, **o**, **t**, **y** depict neurons with anti-FMRFamide. Stacks of confocal micrographs of actin labeling are displayed at two different depths in the body to show both muscles and gut cavity organization. **a**–**e** are control larvae exposed to 0.2% DMSO during the interval between the 4 cell stage and early gastrula. **f**–**y** are larvae resulting from exposure to 20 μM SB431542. **f**–**j** are of a larva resulting from exposure during the interval between the 4 and 32 cell stage. **k**–**o** are of a larva resulting from exposure during the interval between the 16 and 32 cell stage. **p**–**t** are of a larva resulting from exposure during the interval between the 32 cell stage and late cleavage stage embryo. **u**–**y** are of a larva resulting from exposure during the interval between the late cleavage and early gastrula stage. Anterior is to the left in all panels. al: abnormal lumen; at: apical tuft attachment point; black arrows: cilia; cc: circumesophageal connective; cbr: cerebral commissure; dor: dorsal; hg: hindgut; mc, main connective; mg: midgut; sc: subesophageal commissure; st: stomodeum; white arrowheads: dorsal longitudinal muscle fibers; white arrows: disorganized muscle fibers; yellow arrows, disorganized neuronal processes

A defect in dorsal–ventral axis formation occurs when embryos are exposed to SB431542 during a restricted time interval (Fig. 5). Control embryos exposed to 0.2% DMSO during the 4 cell to early gastrula stage result in 52/53 phenotypically normal larvae with differentiated cell types and three clearly distinguishable body axes (n = 3 technical replicates) (Fig. 5a–e). In contrast, embryos exposed to 20 μM SB431542 during the interval between the 4 and 32 cell stage result in severely abnormal larvae that have differentiated cell types and a circular morphology (n = 4 technical replicates) (Fig. 5f–j). In general, abnormal larvae are missing an identifiable stomodeum, midgut, and hindgut (Fig. 5f, g, i). However, larvae do possess a small abnormal internal lumen in the trunk (Fig. 5i; al). The number of actin fibers is reduced and the fibers present have a disorganized arrangement (Fig. 5h; white arrows). There is no detectable apical tuft attachment point (Fig. 5h). Although there are a few neuronal processes, these are disorganized (Fig. 5j; yellow arrows) and there is no identifiable cerebral commissure, subesophageal commissure, circumesophageal nerve or main connective nerves. As such, all three body axes were detectable in only 11% (*n* = 6/56) of larvae and were undetectable in 89% of cases (*n* = 50/56).

Embryos exposed to 20 μM SB431542 during the interval between the 16–32 cell stage resulted in abnormal larvae, with elliptical morphology and differentiated cell types (*n* = 3 technical replicates) (Fig. 5k–o). Larvae are missing an identifiable stomodeum, midgut, and hindgut (Fig. 5k, l, n), but do possess an abnormal gastric lumen within the larval trunk (Fig. 5n; al). There are numerous actin fibers present that are highly disorganized (Fig. 5m; white arrows), and there is no detectable apical tuft attachment point. A cerebral commissure is detectable in these larvae indicating anterior polarity (Fig. 5o; cbr); however, the other anti-FMRFamide immunoreactive neuronal processes present in the trunk are disorganized (Fig. 5o; yellow arrows). All three body axes are detectable in 14% (*n* = 6/44) of larvae and undetectable in 86% (*n* = 38/44) of larvae.

Embryos exposed to 20 μM SB431542 during the interval between the 32 cell to late cleavage stage result in abnormal larvae with differentiated cell types (*n* = 3 technical replicates) (Fig. 5p–t). These larvae have an elliptical shape (Fig. 5p). An abnormal stomodeum opening is detected by DIC optics, and through nuclear and actin staining (Fig. 5p, q, s). Larvae also possess an abnormal gastric lumen within the larval trunk (Fig. 5q and s). There is no detectable apical tuft attachment point (Fig. 5r). Most of the actin fibers present are disorganized (Fig. 5r; white arrows); however, a partial pair of dorsal longitudinal muscles is detectable (Fig. 5r; white arrowheads). The cerebral commissure and main connectives are present and show bilateral symmetry (Fig. 5t). Altogether, these anti-FMRFamide immunoreactive components indicate anterior identity by the presence of the cerebral commissure as well as bilateral symmetry via the bilateral neuronal processes. Bilateral symmetry is further indicated via the anterior-most portion of the pair dorsal longitudinal muscles (Fig. 5r). A dorsal–ventral axis is present as evidenced by the combination of the dorsal longitudinal muscles on the opposite face of the larvae in relation to the stomodeum opening and the FMRFamide immunoreactive neuronal processes. As such, all three body axes are detectable 89% (*n* = 35/39) of resulting larvae, and undetectable in 10% (*n* = 4/39).

Last, embryos exposed to 20 μM SB431542 during the interval between the late cleavage and early gastrula stage result in the least phenotypically abnormal larvae of all the time intervals tested (*n* = 3 technical replicates) (Fig. 5u–y). The overall shape of these larvae resembles wild-type larvae (Fig. 5u). That is, they are elongated along one axis, with one end broader in width relative to the other end, and the widest part of the body is positioned approximately half way along the longest axis. In the gut, distinct stomodeum, midgut, and hindgut compartments are detectable, although the gut is not completely normal (Fig. 5v, x). An apical tuft attachment point (Fig. 5w), cerebral commissure (Fig. 5y), and bilateral main connectives (Fig. 5y) are all present. Some of the actin fibers present are disorganized (Fig. 5w; white arrows); however, in the anterior-most portion of the body there is a pair of dorsal longitudinal muscles (Fig. 5w; white arrowheads). These altogether indicate anterior identity via presence of the cerebral commissure (Fig. 5y) and apical tuft attachment point (Fig. 5w). The apical tuft attachment point and the partial formation of the dorsal longitudinal muscles indicate dorsal identity. The stomodeum opening and main neuronal connectives likewise indicate ventral identity. Bilateral symmetry is visible via the presence of a bilateral pair of main connectives and of dorsal longitudinal muscles in the anterior portion of the body. For this time interval, all three body axes are detectable in 96% (*n* = 48/50) of larvae and undetectable in only 4% (*n* = 2/50) of cases.

Experimental and control conditions were compared using an omnibus Chi square test of homogeneity followed by post-hoc pairwise comparisons using a *z* test of two proportions. The results of these tests indicate that exposures during the 4–32 cell stage and the 16–32 cell stage are not significantly different from each other (*p* > 0.05). However, both conditions result in a significantly lower proportion (*p* < 0.05) of larvae with a detectable dorsal–ventral axis when compared to larvae of the DMSO control condition, the 32 cell to late cleavage stage condition, or the late cleavage to early gastrula stage condition. Conversely, exposures during the 32 cell to late cleavage stage and the late cleavage to early gastrula stage are not statistically significantly different from each other (*p* > 0.05), nor from animals in the DMSO control condition. These data suggest that signaling via the Activin/Nodal pathway functions in dorsal–ventral axis formation prior to the 32 cell stage, likely completing its organizing activity at the end of the 16 cell stage.

## Discussion

### The D quadrant organizing activity is completed prior to the end of the 32 cell stage and involvement of the Activin/Nodal pathway is implicated

We investigated the identity of the *C. pergamentaceus* organizing signal and its timing of action via short embryonic exposures to chemical inhibitors during different developmental time intervals. Chemical interference of the Activin/Nodal pathway but not the BMP or MAPK pathway results in larvae that lack a detectable dorsal–ventral axis. It will be important that future studies use functional genomic knockdown approaches to demonstrate the role of Activin/Nodal signaling in dorsal–ventral patterning to verify the specificity of SB431542. We also identified a narrow time interval during which axis specification occurs. Inhibition of Activin/Nodal signaling using SB431542 results in a loss of all detectable body axes in larvae resulting from treatment during the 4–32 cell stage. In treatments during the 16–32 cell stage, an anterior–posterior axis is detectable; however, bilateral symmetry and a dorsal–ventral axis are not distinguishable. In contrast, in larvae resulting from treatment during either the 32 cell to late cleavage stage or between the late cleavage and early gastrula stage, all three body axes are identifiable. As such, it is unlikely that these latter time intervals are involved in organizing activity. Altogether, these data provide experimental evidence suggesting that organizing activity in *C. pergamentaceus* encompasses the 16 cell stage and is completed prior to the end of the 32 cell stage.

In all cases thus far for spiralians, the cell with organizing activity is localized to a descendant of the D quadrant. The D quadrant of *C. pergamentaceus* fits this pattern since it has been experimentally demonstrated to have organizing abilities [27, 31]. Future investigations could seek to conduct single cell ablations, whereby individual cells of the D quadrant between the 8 and 32 cell stages are systematically ablated to determine the cellular identity of the *C. pergamentaceus* organizer. We hypothesize that the organizing signal is localized to micromere 2d at the 16 cell stage as is the case for *C. teleta* [11].

The results of this study suggest that Activin/Nodal signaling is involved in establishing the dorsal–ventral body axis in *C. pergamentaceus,* and that organizing activity is completed during the 16 cell stage, when the second quartet of micromeres is present. Our data further indicate that there are similarities regarding the timing of organizing activity between *C. pergamentaceus* and *C. teleta,* two annelids that undergo unequal spiral cleavage yet occupy very different positions in the annelid phylogeny. In both annelids, organizing activity occurs one to two cell divisions earlier than observed in several mollusks in which organizing activity is localized to either cell 3D or 4d (reviewed in [50]). The differences in timing across species mean that in different animals, organizing activity is localized to different cells that have distinct descendant fates. For example, 3D, the cell with organizing activity in several mollusks, generates endoderm and trunk mesoderm. In contrast, the cell with organizing activity in *C. teleta*, 2d, generates all of the ectoderm of the trunk and pygidium [51]. It will be important in future studies to determine the fate of 2d in *C. pergamentaceus* using intracellular lineage tracers, although general conservation in the fate of 2d across annelids suggests that 2d in *C. pergamentaceus* will also give rise to trunk ectoderm [10, 52, 53].

### Variation in spiralian axes patterning signals

Investigations into the molecular signals that mediate organizing activity in spiralians have revealed varying roles for signaling pathways of the TGF-β superfamily and the ERK/MAPK signaling pathway. For example, in the polychaete *C. teleta,* signaling via the Activin/Nodal branch of the TGF-β superfamily pathway, not the BMP branch, is the primary dorsal–ventral axis patterning signal [38, 39]. Like in *C. pergamentaceus,* embryonic exposure to the Activin/Nodal chemical inhibitor SB431542 during the time of organizer activity in *C. teleta* results in larvae lacking bilateral symmetry and a detectable dorsal–ventral axis [38]. These larvae are phenotypically similar to those resulting from ablations of organizer cell 2d [11]. While wild-type *C. teleta* larvae at stage 6 are elongated along the anterior–posterior axis, have an anterior and posterior ciliary band, a bilaterally symmetric central nervous system in the head and trunk, a ventral mouth and a clear dorsal–ventral axis; SB431542-treated and 2d-ablated animals are spherical, have a single ciliary band, possess radialized neuronal head features, and have reduced specification of the trunk [11, 38]. Activin/Nodal was further confirmed to function in *C. teleta* dorsal–ventral patterning by morpholino knockdown of *Ct*-*Smad2/3* and *Ct*-*Smad1/5/8,* transcription factors specific to the Activin/Nodal and BMP pathways, respectively [39]. These resulted in a significant loss of dorsal–ventral patterning in *Ct*-*Smad2/3* morphants but not in *Ct*-*Smad1/5/8* morphants [39]. Furthermore, *Smad2/3* MO morphants possess a similar but less severe phenotype than larvae resulting from 2d ablations or SB431542 exposures. For instance, *Smad2/3* morphants have radialized head features, but a greater proportion of morphants have more defined trunk structures, despite lacking dorsal–ventral patterning [39].

In contrast to its conserved role in dorsal–ventral patterning of many bilaterians, the BMP signaling pathway does not appear to mediate dorsal–ventral axis patterning in some spiralians. In this study, exposures to the BMP inhibitors DMH1 and dorsomorphin dihydrochloride during multiple early developmental time intervals did not elicit any phenotypic effects in *C. pergamentaceus*. This could suggest that BMP signaling is not involved in the development of any of the morphological features analyzed; however, it is also possible that these inhibitors are ineffective in *C. pergamentaceus.* Differences in the molecular architecture of the targets of DMH1 (ALK 2) and dorsomorphin dihydrochloride (ALK 2 and ALK 3) could account for the lack of effectivity in *C. pergamentaceus*, despite eliciting reproducible phenotypes in several other spiralian species. For instance, in *C. teleta*, DHM1 exposures do not elicit any phenotypic effects; however, embryonic exposures to dorsomorphin dihydrochloride result in abnormal larvae, which maintain their normal body axes [38]. The MO knockdown of *Ct*-*Smad1/5/8,* a transcription factor specific to the BMP pathway, further supports this finding in *C. teleta*. In these experiments, larvae resulting from zygotic injection of *Smad1/5/8* MO result in a phenotype similar to those resulting from treatment with dorsomorphin dihydrochloride, and the dorsal–ventral axis is clearly distinguishable [39]. A recent investigation in the slipper snail *Crepidula fornicata* similarly demonstrated that BMP signaling does not appear to play a role in organizer function [54]. In *C. fornicata*, ectopic addition of recombinant BMP4 protein or the inhibition of BMP signaling by exposure to DMH1 results in larvae with normal body axes in the trunk. Embryonic exposure to DMH1 does, however, result in head defects [54].

In contrast, BMP signaling has been implicated in dorsal–ventral axis patterning of other spiralians. For example, although brachiopods do not develop according to a stereotyped spiral cleavage program [8], BMP signaling affects embryonic dorsal–ventral patterning. In *Novocrania anomala* and *Terebratalia transversa*, continuous exposure to the BMP inhibitor DMH1 between the 2 cell stage and early larval stages causes the expression of ventral ectodermal markers to expand dorsally [55]. Similarly, in early-stage embryos of the oyster *Crassostrea gigas,* the ligand and its antagonist, BMP2/4 and Chordin, are expressed in restricted and complementary patterns along the dorsal–ventral axis, and were hypothesized to function antagonistically to each other during dorsal–ventral patterning [56]. Support for this hypothesis comes from embryonic exposures to dorsomorphin dihydrochloride, which result in expansion of ventral gene expression dorsally in gastrula stage animals, while dorsal gene expression is abnormally restricted [37].

Interestingly, in *Trita obsoleta*, both the ERK/MAPK and BMP pathways appear to function in organizing activity. Ablation experiments in *T. obsoleta* have demonstrated that the D quadrant macromere possesses organizing capabilities through the 32 cell stage and that the organizing activity is localized to cell 3D [12, 15, 16]. Ablation of 3D results in larvae that are missing structures not directly descended from the D quadrant, implying the need for an inductive, axial patterning signal. Investigations into the *T. obsoleta* organizing signal revealed that the inhibition of MAPK signaling by U0126 results in larvae phenotypically similar to those following D quadrant ablations [17]. Further investigations revealed that morpholino knockdown of *loDpp*, a ligand in the BMP signaling pathway, also results in larvae that are phenotypically similar to those larvae resulting from embryonic ablations of the D quadrant or exposure to U0126 [36]. Since both the ERK/MAPK and BMP pathways are implicated in *T. obsoleta* organizing activity, it is possible that the receptors function using non-canonical signaling via the MAPK pathway [57, 58].

Activation of the MAPK pathway has also been linked to organizing activity in the limpet *Tectura scutum*, and activated MAPK is detectable in macromere 3D at the 32 cell stage [14]. Inhibiting MAPK activation with U0126 prevents the differentiation of 3D and reveals a role for MAPK in inducing the fates of surrounding cells and facilitating normal axis specification. In the gastropod *Haliotis asinina,* activated MAPK is also detectable in macromere 3D [34]. Embryonic exposures to U0126 prevent the activation of MAPK in macromere 3D and result in abnormal trochophore larvae. In these larvae, however, the dorsal–ventral expression of ectodermal genes is only affected in the head, but not in the posttrochal (trunk) region [34]. This leaves open the possibility that another pathway is involved in *H. asinina* dorsal–ventral patterning. From these limited investigations and the findings of this study, it is evident that the molecular pathways orchestrating axis patterning vary even within the Spiralia.

### *Chaetopterus pergamentaceus* gut and muscle development is affected by MAPK inhibition

The results presented in this study demonstrate that unlike some of the aforementioned spiralians, the MAPK signaling pathway does not play a role in *C. pergamentaceus* dorsal–ventral axis formation. Instead, larvae resulting from treatment with the MAPK inhibitor U0126 during both the 4 cell to late cleavage stage and the late cleavage stage to early gastrula time intervals exhibit abnormal muscle and gut development. In particular, larvae exhibit a disorganization and reduction in the number of muscle fibers. A similar phenotype is also observed in the annelids *P. dumerilii, Alitta virens,* and *C. teleta.* In these species, inhibition of the ERK/MAPK pathway through embryonic exposure to U0126 results in several defects, but does not affect dorsal–ventral axis patterning [11, 35, 59]. In *P. dumerilii*, MAPK is activated during gastrulation. Inhibition of MAPK signaling with U0126 during gastrulation results in larvae that have a reduced number of muscles, and a disorganized muscle pattern [35]. Similarly, in *C. teleta,* larvae resulting from embryonic treatments with U0126 have a decreased number of muscle fibers [11]. In *A. virens,* the differentiation and morphogenesis of the mesoderm are similarly reliant on MAPK signaling since early embryonic treatment with U0126 disrupts the spatial organization of mesodermal genes [59]. In addition to exhibiting a muscle phenotype, *C. pergamentaceus* larvae resulting from embryonic exposure to U0126 lack a detectable tripartite gut. Instead, these larvae possess a stomodeum and an abnormal gut cavity. Moreover, larvae resulting from exposures during the late cleavage to early gastrula time interval present a slightly more severe gut phenotype. In *C. teleta,* larvae resulting from embryonic treatments with U0126 also exhibited gut abnormalities in the form of a reduced foregut [11]. Altogether, the similarities in phenotype shared among these four annelids strongly suggest that in Annelida, the ERK/MAPK pathway functions in muscle and gut development.

## Conclusions

Across Bilateria, embryonic dorsal–ventral axis patterning has widely been attributed to BMP signaling [60]; however, there now exist a growing number of examples, particularly within the Spiralia, demonstrating that there is more molecular diversity in dorsal–ventral patterning than was previously appreciated. Our findings here suggest a second instance in which Activin/Nodal signaling functions in dorsal–ventral axis patterning within Spiralia. The data from this study together with results in *C. teleta* suggest that the ancestral state of annelid dorsal–ventral axis patterning involved Activin/Nodal signaling, although further sampling within annelids will be necessary to strengthen this argument.

Both the Activin/Nodal and BMP pathways have been implicated in dorsal–ventral axis patterning in different members of the Spiralia. Since these signaling pathways are branches within the TGF-β superfamily, it is intriguing to consider that the ancestral mode of dorsal–ventral axis patterning in spiralians utilized both branches. In this scenario, molecular evolutionary changes would lead to loss of function of one branch of the signaling pathway to induce the dorsal–ventral body axis in some animal lineages, whereas in other lineages, loss of function would occur in the other branch of the signaling pathway. Thus, one would expect either BMP or Activin/Nodal signaling to be utilized throughout the Spiralia. Furthermore, the phylogenetic node(s) at which these genetic changes occurred would dictate the exact pattern of which pathway is utilized. For example, there may be an annelid-specific pattern. Alternatively, if there were multiple independent evolutionary changes within either annelids or mollusks, each of these clades may contain species that utilize Activin/Nodal or BMP signaling to mediate organizing activity. One hint of possible intraclade variation comes from comparisons of *C. teleta* and *C. pergamentaceus* with that of the annelid *Hydroides hexagonus.* The activation of MAPK in 4d in *H. hexagonus* led to the proposal that 4d is the embryonic organizer in this annelid, and if this is the case, it hints variation in both the identity and possibly molecular signaling of the embryonic organizer within annelids [14]. The molecular differences across species would most likely be regulatory changes rather than loss of genes from the genome since both signaling pathways have pleiotropic roles during development.

To determine the extent of variability in the molecular signal that mediates organizing activity within Spiralia, and reconstruct the ancestral state for the embryonic organizer, future investigations should seek to sample other animals. To our knowledge, experimental investigations into the molecular signal orchestrating organizing activity have only been conducted in two annelids, *C. teleta* [38, 39] and *C. pergamentaceus*. As such, it will be of particular interest to sample other annelids such as members from the Errantia clade, as well as from other early branching annelid taxa such as the Sipuncula. Though current phylogenomic studies show a clear alliance of sipunculids with annelids (reviewed in [61]); historically, a close phylogenetic affinity between sipunculids and mollusks was argued based upon the shared presence of a ‘molluscan cross’ [62]. The molluscan cross refers to a particular arrangement of micromeres during early cleavage stages in some mollusks, and contrasts with a different arrangement of micromeres observed in some annelids and known as the ‘annelid cross’ [43, 63]. The strong concordance in phenotype between the chemical inhibition and morpholino knockdown studies in *C. teleta* suggests that it may be possible to substantially increase our sampling with commercially available chemical inhibitors [38, 39]. Of course, other spiralian phyla such as the equal cleaving Nemertea should also be investigated. Only with increased sampling will we be able to understand and reconcile the relationship between the high conservation in the cleavage program and fate map of many spiralian taxa with variation in the molecular signals utilized during early development and formation of diverse body plans of the Spiralia.

## Supplementary information

**Additional file 1: Fig.** **S1.** Early embryonic exposure to DMH1 or dorsomorphin dihydrochloride does not affect larval morphology. Each column contains merged confocal-stacked images of a single L2 larva. Each row depicts labeling for nuclei with Hoechst, actin filaments with phalloidin, or neurons with an anti-FMRFamide antibody. Stacks of confocal micrographs of actin staining are displayed at two different depths in the body to show muscle and the tripartite gut. Panels A–D are control larvae exposed to 0.2% DMSO during the interval between the 4 cell stage and early gastrula. Panels E–L are larvae exposed to 20 μM DMH1. Panels E–H depict a larva resulting from exposure during the 4 cell to late cleavage stage. Panels I–L depict a larva resulting from exposure during the late cleavage and early gastrula stage. Panels M–T are larvae exposed to 20 μM dorsomorphin dihydrochloride. Panels M–P are of a larva resulting from exposure during the 4 cell to late cleavage stage. Panels Q–T are of a larva resulting from exposure during the late cleavage and to early gastrula stage. All panels are dorsal views with anterior to the left. Abbreviations: at, apical tuft attachment point; cc, circumesophageal connective; cbr, cerebral commissure; dor, dorsal; hg, hindgut; mc, main connective; mg, midgut; sc, subesophageal commissure; st, stomodeum; white arrowheads, dorsal longitudinal muscle fibers.

## Data Availability

All data analyzed or generated in this study are included in this manuscript
