# Supplementary figures and images for: Activin/Nodal signaling mediates dorsal–ventral axis formation before third quartet formation in embryos of the annelid Chaetopterus pergamentaceus
Source: EvoDevo. 2020 Aug 10;11:17. doi: 10.1186/s13227-020-00161-y (PMC7418201; doi:10.1186/s13227-020-00161-y)

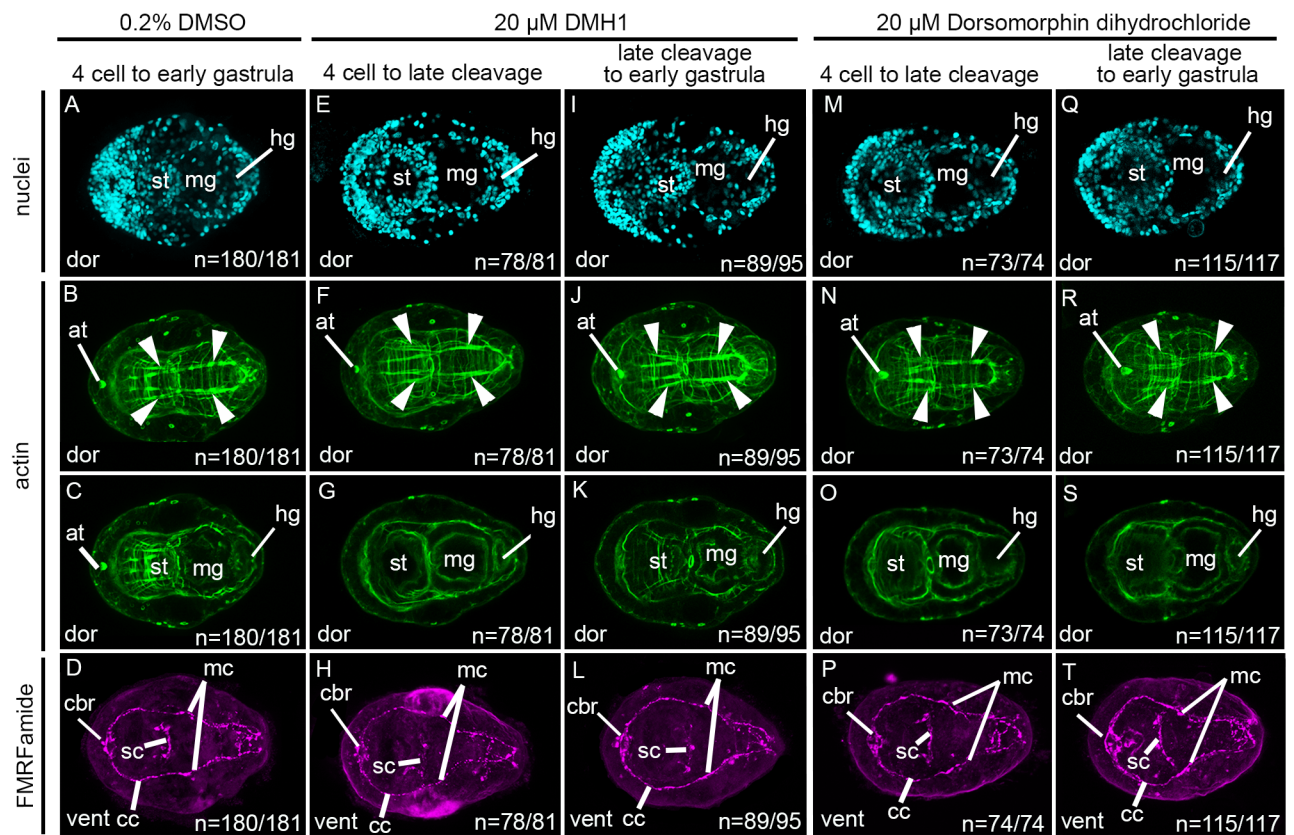

Supplement: Supplementary file 1 — Additional file 1: Fig. S1. Early embryonic exposure to DMH1 or dorsomorphin dihydrochloride does not affect larval morphology. Each column contains merged confocal-stacked images of a single L2 larva. Each row depicts labeling for nuclei with Hoechst, actin filaments with phalloidin, or neurons with an anti-FMRFamide antibody. Stacks of confocal micrographs of actin staining are displayed at two different depths in the body to show muscle and the tripartite gut. Panels A–D are control larvae exposed to 0.2% DMSO during the interval between the 4 cell stage and early gastrula. Panels E–L are larvae exposed to 20 μM DMH1. Panels E–H depict a larva resulting from exposure during the 4 cell to late cleavage stage. Panels I–L depict a larva resulting from exposure during the late cleavage and early gastrula stage. Panels M–T are larvae exposed to 20 μM dorsomorphin dihydrochloride. Panels M–P are of a larva resulting from exposure during the 4 cell to late cleavage stage. Panels Q–T are of a larva resulting from exposure during the late cleavage and to early gastrula stage. All panels are dorsal views with anterior to the left. Abbreviations: at, apical tuft attachment point; cc, circumesophageal connective; cbr, cerebral commissure; dor, dorsal; hg, hindgut; mc, main connective; mg, midgut; sc, subesophageal commissure; st, stomodeum; white arrowheads, dorsal longitudinal muscle fibers. [file 13227_2020_161_MOESM1_ESM.pdf]
